# Special Pathology and Therapeutics, Founded on Clinical Observation

**Published:** 1842-04

**Authors:** 


### Art. III.

*Die Specielle Pathologie und Therapie vom Klinischen Standpuncte ausbearbeitet.* Von Dr. Carl Canstatt, Königlich-bayerischem Gerichtsarzt, und Mitgliede meherer gelehrter Gesellschaften. Erster Band.—*Erlangen*, 1841. 8vo, pp. 331.

*Special Pathology and Therapeutics, founded on Clinical Observation.* By Dr. Charles Canstatt. First Vol.—*Erlangen*, 1841.

It is not our intention to give a systematic and complete analysis of the volume before us, worthy though it be of a somewhat detailed examination. We shall confine our remarks to such only of its contents as seem most likely to interest the English reader.

In his preface, Dr. Canstatt remarks that clinical research may be prosecuted on one of two methods, the *Morphological* and the *Genetic* or

*Historical.* The one is founded on the particular form (μορφή) or appearance under which disease manifests itself; the other on a consideration of the causes and morbid processes of which that disease is the result. "The latter method," he observes, "just awakening from the long lethargy into which the exclusive pursuit of pathological anatomy had laid it, is, notwithstanding its high importance, as yet but in its infancy." Further on, the author refers to the rapid progress which neuro-physiology is making, and must ever make in comparison of its "sister science," neuro-pathology; to the former of which, the latter must be content to act the part of a mere handmaid.

There is, doubtless, truth in these remarks. If we enquire to what extent practice has been advanced by pathological anatomy, we shall find it to be less than what might at first be expected. In other words, the superiority, as regards treatment, of physicians of the present time over their predecessors of earlier ages is relatively less than the superiority of the former in pathological acquirement is great. For example, our minuter, and, if we may be allowed the expression, our microscopic acquaintance with the phenomena of inflammation has immaterially, if at all, influenced the treatment of that disease; and the same may be said of fever and several other principal maladies. The information, moreover, which pathological anatomy can convey is often far from final. Thus, the phthisical disorganization of the lungs is not, properly speaking, the cause of death; it is rather the impediment to the performance of the function of respiration, and its various consequences produced by the disorganization referred to, which bring about the fatal result. Now, on lesions such as this, pathological anatomy throws little or no light. It is to be added, that towards the elucidation of the nature of many diseases, morbid dissection has already done all that it can be expected ever to do: so that a great number of the elaborate *post-mortem* examinations, and subsequent verbose reports and comments with which the pages of medical journals are often needlessly loaded—consisting as they do of things a thousand times observed, and as often described—are rather for the advantage of the individuals who undertake them than for that of the public or the profession. We are led to these remarks from no wish, the most remote, to disparage pathological anatomy or underrate its just claims, but partly because the observation of Dr. Canstatt naturally suggested them; partly because this branch of our art is still cultivated and written of by many, as if it were the end, not a mere means of helping us to a knowledge of the causes by which structural changes are produced. We even venture to lay it down as an axiom that just in proportion as we could arrive at a knowledge of disease, independently of pathological anatomy, that is, before the occurrence of those more palpable changes of which it is the province of pathological anatomy to make us cognizant, the nearer should we be to a knowledge of the essence of disease, and of those stages of it in which remedial measures are most likely to avail.

Then, as to the author's other remark, that neuro-physiology and physiology in every department must precede the respective pathologies, it scarcely requires the slightest illustration. It is obviously first necessary to ascertain what is the normal law, before we pretend to decide wherein aberrations from that law consist. Health, like social order, is the rule; disease, like anarchy, the exception: and consequently we must first possess our minds with clear conceptions of what the primary and proper order and course of things are, before, in the event of these being interrupted, we can distinctly understand what is the desirable state of matters which it should be our aim to restore.

In the work before us, disease is treated of after the morphological method. The volume consists of twenty-two sections, relating to the following subjects: Atrophy; Hypertrophy; Plethora; Anemia; Chlorosis; Congestion; Hyperemia; Inflammation; Hemorrhagy; Anomalies of the Secretions; Dropsies; Pneumatoses; Obesity; Homœoplastic Formations; Pseudo-plastic Formations; Tuberculosis and Scrofulosis; Lithiasis; Malaria; Ossification (or hardening, in general, of tissues); Fever; Neuroses. Many of these subjects are subdivided, and a number of important diseases, not named in the above list, are treated of, as species of the genera now enumerated. In a second volume, Dr. Canstatt contemplates an exposition of Clinical Medicine on the Genetic or Historical plan; which is more applicable to diseases of the system (individualität), such as, the cachexies and dyschymoses; and also to cosmical diseases, or those dependent on soil, climate, or particular atmospheric constitution, as for example, endemics and epidemics. After disease has localized itself in some particular part or organ, and become overt or, as the author expresses it, formal, the other or morphological plan is to be adopted; although even then a study of the malady, on the genetic principle, is, in a therapeutical point of view, highly useful, in order that we may ascertain the causal indication, ever most important for the right apprehension and radical treatment of disease.

HYPERTROPHY. In the section on hypertrophy (p. 3,) Dr. Canstatt observes, "all organs and tissues consist merely of vessel, blood, and nerve, and formative matter. The blood is the fluid material; the formative matter is the condensed and condensing plasma in the tissue which is forming. . . . The product of the nutrient process is in the concrete tissue, neither blood, nor vessel, nor nerve, but the condensed and condensing plasma." This peculiar and somewhat oracular language, which, be it remarked, is that of other German physiologists, would lead us to infer that the plasma is a substance distinct from the blood and intermediate between it and the already organized tissues, vessel, and nerve; that it is neither albumen nor fibrine, nor yet a compound of these; that the plasma is first constituted separately, and that then vessel, nerve, &c., derive from it their particular molecular conglomeration. Now this is a purely gratuitous supposition It removes no difficulty as to the manner of the vegetative process; since that the elective attraction of homogeneous matter, by the already organized tissues should take place from the plasma is just as mysterious as that it should take place from the blood directly; while the supposition of an intermediate, independent product, the plasma, seems only to render the process of organic formation more circuitous and perplexing. Indeed at page 6, Dr. Canstatt himself appears to adopt the more ordinary and simple view. "The elucidation," he says, "of the phenomena of nutrition is impossible, so long as we allow not to every organ, to every organic element, to every organo-crystallizable particle, specific powers of assimilation, whereby each of these can operate on the nutrient materials *contained in the blood;* can appropriate these to its own particular augmentation." There is no mention here of the plasma; and it is singular that Dr. Canstatt should employ this term elsewhere, yet omit all reference to it in this, a circumstantial statement of the nutrient process. We believe that Müller's account of the mode of nutrition is the one generally received, and is, perhaps, as explicit and precise as the present state of our knowledge admits of. "Nutrition must be effected through the coats of the capillary vessels, and the process consists in the fluid parts of the blood permeating the parietes of the capillaries, while the solid particles are visibly carried onwards into the veins. The most important materials for nutrition are the albumen and fibrine dissolved in the liquor sanguinis." (Baly's Müller's Elements of Physiology, vol. i. p. 360.)

In discoursing of hypertrophy (p. 6), the author compares the eccentric attraction of the individual tissue or organ in which that morbid process is going on, to the conglomerative growth of a crystallizable salt in a salt-ley, by which the constituents of the saline mass which are in solution are led to deposit themselves on the nucleus. He supposes that in a similar manner, the organized tissue, the seat of an excessive trophical action, attracts from the "blood-stream" formative matter appropriate to it. It strikes us that the illustration, without really enlightening our conceptions of the vegetative process, is too mechanical in its character to be legitimately paralleled with that of animal nutrition. The author recognizes as one cause of hypertrophy, a remissness in the absorbent action of the part, which our readers are aware is doubted of by other physiologists; although, in our opinion, on no good grounds.

Dr. Canstatt's views of what may be expected from treatment in hypertrophy are, in the last degree, desponding. Hypertrophic action is, he conceives, so nearly connected with that of normal nutrition, that we cannot exert any influence on the former without debilitating the entire latter process; as, on the other hand, we cannot suffer physiological nutrition to go on uninterfered with, without encouraging the morbid action. "The cure by hunger," he observes drily, "may be resorted to in desperate cases." Valsalva's decisive and successful treatment on this plan, in cases of aneurism, which, in certain of its characters, and in the therapeutic means proper to it, somewhat resembles hypertrophy, will be in the recollection of our readers. It must at the same time be remembered, in regard to aneurism, that severe abstinence may accelerate it, by attenuating and debilitating the tissues, and lessening the resistance which they would otherwise oppose to the dilating force of the current of blood. Still, in cases of hypertrophy, we have no small faith in a judiciously employed system of abstinence: or to express ourselves more correctly, in a moderate reduction of the wonted amount of nutritive supply. We think that the author is in error in supposing, at least in all cases, that abstinence affects equally parts which are normally developed, and those that are morbidly so. This is not always, we may say, is not usually the case, so long as there is mere hypertrophy, not structural change or pseudo-plastic formation, and so long as the constitution is in any considerable degree sound. In such cases, abstinence affects, first and chiefly, the morbid growth. Nature gives up, in the first instance, that which is extraneous and parasitical, in preference to what is normal both in structure and in degree of development. This is an important law, observable in a thousand cases, without the auspicious operation of which, indeed, one half of our therapeutic labours would be vain.

As an internal means, iodine is suggested; but then the author sceptically surmises that this is a means dangerous to be had recourse to, since the modus operandi of iodine consists in undermining the universal process of nutrition. It is not to be doubted that, in some measure, it does so; still the same reasoning is applicable to it as to abstinence, namely, that simple hypertrophied parts are taken down previously to normally developed organs being affected by it. No mention is made of antimonials, which, however, as well as nitre, are worthy of attention. Bloodletting, particularly when it can be brought to bear locally on the organ, which is the seat of hypertrophic action, may be useful; but even in regard to it, the author is far from sanguine, since if practised in moderation, it is apt to prove fruitless; if energetically employed, it is more likely to promote anemia, dropsy, and debility, than to cure the hypertrophy. He entertains the same opinion of purgatives. Attempts to arrest the hypertrophy of glandular organs, by augmenting their secretions may, in some cases, do good; but on the other hand, the excitement produced in the endeavour to stimulate the organ to more active secretion may but help to augment and accelerate the hypertrophy.

Dr. Canstatt considers powerful revulsives as among the most efficient modes of treatment; a seton, for example, in hypertrophy of the heart. As later means, tying of the principal blood-vessels, or section of the chief nerves of the part, or extirpation in the case of organs that may be spared, as the thyroid and mammary glands, the testicles, &c. may be resorted to. He justly regards the causal indication as most important, where it can be ascertained. It is singular that the author should omit all reference to sedatives as very powerful indirect means of checking that species of hypertrophy dependent on excess of innervation, and consequently of increased and sthenic action. We have noticed unequivocal benefit from the prolonged use of hyoscyamus, hydrocyanic acid, opium, and other sedatives in cases of hypertrophy of the heart, other means being conjointly employed.

We pass over the section on ATROPHY. Many or most of the causes of atrophy are suggested by those of hypertrophy. Thus atrophic and hypertrophic affections dependent on the antagonism or sympathy of related organs, mutually produce each other. Wasting of one kidney gives rise to increased action, to hypertrophy of the other; and, *vice versâ*, converse results ensue. PLETHORA we also omit; a lesion which, Dr. Canstatt observes, is to the fluid what hypertrophy is to the solid constituents of the body. We may remark that, according to the author, hypertrophy always begins in the plasma; and consists in this substance augmenting itself in morbid disproportion to the vascular and nervous tissues of the part; and that therein lies the distinction between pathological and physiological nutrition. In like manner, plethora is not the result of a simultaneous increase of all the elements of the blood, but of some one of them. There may consequently be cacho-chymious, lymphatic, venous, adipose, serous, and plastic plethora.

ANEMIA. The nature and appearances, the causes, course, and cure of anemia, or, as Dr. Canstatt more properly designates it, oligemia, next occupy attention. This is a disease in which, according to the author, medical art is more efficient than in most others, and "reaps," to use his phrase, "the most beautiful triumphs." (p. 31.) The remark may be true in regard to oligemia resulting from *some* of the causes enumerated by the author, those, namely, dependent on "an unusually increased consumption of the blood;" as by inordinate purgation, bloodletting, lactation, salivation, purulent discharges, diarrhœa, sensual excesses, pregnancy. But the same thing cannot be alleged of anemia caused by others of the derangements referred to by him, such as diseases of the spleen, lungs, heart, mesenteric glands—the cure of which forms of the affection, depending of necessity on the prior removal of the above formidable maladies, must either be altogether beyond the reach of art, or else require very protracted treatment.

Chlorosis, Dr. Canstatt regards as "a spasmodic form of oligemia," (p. 32;) and as his opinions of the nature of this disease, and of the treatment which it demands, tend to simplification by showing chlorosis to be altogether identical with anemia, we shall briefly notice them. "We have to choose," he observes, "whether to range chlorosis among the dyscrasiæ, or to treat of it in this place and in connexion with anemia. We prefer the latter, since, in fact, anemia and chlorosis, in their symptoms, course, issue, causes, indications of cure, are so entirely identical as to be, in our apprehension, in the strictest sense, one and the same disease. Anemia (or hydremia, oligemia, blood deficient in crasis, however one may choose to designate it,) suffices to explain all the conjoint symptoms of chlorosis, its acrinie (absence of secretion) of the genital organs, its derangement of the digestive function, its nervous affections." (p. 33.)

An asthenic condition of the nervous system is an affection always coincident with the anemia; since, on the one hand, a normal condition of that system is indispensable to a right formation of blood; as, on the other, normal blood is essential to a healthy state of the nervous system. Chlorosis, he thinks, may originate in a faulty state of either of these systems, but in both cases its symptoms, course, and effects are the same. Chlorosis may show itself in lively and sanguineous virgins; and he explains this fact on supposition that the blood of every young woman undergoes, at puberty, certain marked changes, in order that it may be fitted for the manifestation of that peculiar "sensibility and irritability," which belong naturally to the adult female constitution. Rösch supposes that the change which takes place in the blood of girls at this period transmutes it from somewhat of a molluscous into a mammalious character. The blood, which hitherto abounds with albumen, becomes now replenished with fibrine. Dr. Canstatt believes that every young woman suffers more or less considerably from the operation of this change, and that hence, even florid girls may, for a time, exhibit the "qualitative dyscrasies" and other signs of chlorosis.

This disease, when of long duration and severely manifested, may lead to serious consequences, which vary according as the patient is of the phlegmatic or nervous temperament—conducting, in the former, to dropsies; in the latter, to consumption.

The cachectic complexion of the chlorotic patient often leads to a suspicion that she (or he, for young men are incontestably liable to the disease,) is the subject of icteric, hypochondriac, or carcinomatous affections. But the distinction may, with a little care, be easily drawn. In chlorosis, however icteric be the complexion, the conjunctiva retains always its pearly whiteness; while it is first and most affected in icterus. The palpitations and arterial paroxysms and the muscular debility of chlorosis are absent in icterus.

The diagnosis between chlorotic palpitations and those dependent on organic disease of the heart is less easy—since both are characterized by giddiness, wanness, and since chlorosis often actually leads to cardiac disease. The distinction is therefore important. It may be exhibited as follows:

| *Chlorotic Palpitations.* | *Palpitations from Cardiac Disease.* |
|---|---|
| Puberty and female sex predispose. | Are hereditary. [We doubt this.] |
| Come on with other signs of chlorosis or anemia. | Precede the cachectic colour and appearance of the patient. |
| Come on suddenly and depart equally so; have perfect intermissions; are worst after bodily and mental excitement, and at the period when the menses ought to appear. Remit under the measures which relieve the other chlorotic symptoms. | Never depart; but continue always with more or less violence. |
| Stimulants, as iron, relieve. | Stimulants aggravate. |
| Complexion pale, without lividity. | Lips and cheeks livid. |

The author has omitted to add the presence of the anemic murmur, or *bruit de diable*, in the blood-vessels of the chlorotic patient, and the absence of this in the case of disease of the heart.

From the considerable detail, as regards treatment, into which Dr. Canstatt enters, we quote three observations only. The first is, that all the multifarious symptoms of chlorosis do not require separate attention; since depending on a common lesion, to wit, the deficient crasis of the blood, they simultaneously disappear when that fluid is brought to its normal state. Secondly, iron, if not specific in this disease, will at last accomplish a cure in by far the majority of cases: its union with the vegetable bitters ensures this result. The last observation regards the causal indication in chlorosis. This being a disease holding an intimate relation to woman's peculiar sexual constitution and wants, imperatively requires us always to keep in mind the advice of the physician of Cos: "Equidem virginibus suadeo . . . . ut citissime cum viris conjungantur." We believe that while, in many cases of chlorosis, this last prescription is, no doubt, the appropriate one, and will, by itself, prove successful, yet that medicinal and dietetic means will be found efficacious in a large proportion of instances.

Congestion. The congestive orgasm, Dr. Canstatt describes (p. 43,) as consisting "in the manifestation of a power of reaction in the sanguineous system, by which the organic power" (or *selfism*, to render literally the *ichheit* of the author,) "meets any demand upon it, or rouses itself against any hostile influence." The distinction between congestion and hyperemia is somewhat vaguely stated to be, "simply this, that in the former, the action, the movement—in the latter, the inertia, the stationary condition of the organic fluid predominates." (p. 43.) The local and external characters of these two affections greatly resemble. The diagnosis of internal congestion is sometimes extremely difficult. It is occasionally impossible to distinguish between active congestion, passive hyperemia, and actual inflammation, until the fugitive character of the affection proves it to be congestive. Like hyperemia and inflammation, it gives rise to antagonistic and sympathetic symptoms in other organs; and through nervous reflexes and assimetrical distribution of blood, begets chillness, pallor, and anemia of external parts, when deep-lying ones are the seat of the affection.

INFLAMMATION. The section, inflammation, which is ample without being diffuse, contains all the facts and doctrines relating to that morbid process. "So long," the author observes, (p. 56,) "as the reaction against any exciting cause continues moderate, and passes not into an excessive degree, there is congestion. Should the reaction overstep the limits and become inordinate, we say it is hypersthenic and passes into inflammation." Pathological anatomy, he remarks, concerns itself with the change of structure which inflammation causes; clinical science, with the act itself. "In a clinical sense, inflammation begins at the moment of congestion; in an anatomical sense, at the moment of the hyperemia."

The author describes the compound character of inflammation to consist "in an alteration of the local nutrition of the affected part." As this definition confounds, in some measure, inflammation with hypertrophy if not with hyperemia, we prefer, with some late writers, to recognize, as inflammatory, no morbid process which, besides being characterized by heat, swelling, and redness of the part, is not accompanied with *effusion* of some one description or another; generally of coagulable lymph, of pus, or of serum. Whether no action but such as issues in effusion is, rigorously speaking, to be pronounced inflammatory, we shall not now curiously enquire. We only mean to state that, as furnishing a convenient line of demarcation, and preventing ambiguity in pathological discussion, it is best to regard the effusion as an indispensable condition; while no practical ill consequences can possibly result from the adoption of this rule.

The author distinguishes two periods in the inflammatory process, the former of which he subdivides as follows: First. There is the well-known constriction of the capillaries, with a more rapid flow of blood through them. This, he names the period of active congestion, and assigns as its cause reflex action of the vascular nerves, in preference to spasm of the capillaries. (p. 56.) Secondly. Enlargement of the capillary vessels; stagnation and conglomeration of the red globules. This he calls the period of passive hyperemia, attributed, by some, to an increased action of the arteries with a diminished action of the veins; by others, to atony of the capillaries, in which they suppose inflammation essentially consists. Thirdly. Escape from the vessels of serum and red blood, the period of exudation, amalgamation, colliquescence of the tissues. The usual anatomical characters are given with accuracy, and then the author passes on to the "physiological signs," which, he remarks, are those about which the scientific physician principally concerns himself, and which are, by far, the most important. (p. 62.)

Pain, or rather abnormal sensations, (for, as the author justly remarks, the sensations experienced are not necessarily painful,) is the first of these physiological characters. It may, however, be absent when there is inflammation, or be acutely present when there is none; and therefore it, as well as redness and swelling, which may on their part be due to simple hyperemia, is not pathognomonic of inflammation.

To determine the source of augmented temperature in inflamed parts would require a previous knowledge how the physiological heat of the body was maintained; whether it be a product of the respiratory process, or of certain actions in the nervous centres; or of vital interchanges between the solids and fluids; or of some reciprocal influence of the nerves and vessels on each other; or of varying capacities of the blood for caloric. It is to be remarked that the elevated temperature of inflamed parts is not subjective merely, but objective also; that the patient is not only conscious of a greater degree of heat, but that the thermometer, placed against the affected part, indicates a higher temperature.

Nerve and blood are the factors of inflammation as of nutrition, and the morbid process may begin, now in the former and now in the latter. It may originate in the nerves: 1st. By excitement of these, as, for example, when a stimulant agent operates reflexly on the vascular nerves, and, through these, affects the circulation in the capillaries, causing congestion, hyperemia, inflammation; 2d, by paralysis of the nerves, particularly of the sensitive nerves. The vascular nerves thereby lose their necessary stimulus; hence ensue stagnation of the capillary circulation, hyperemia, alteration of nutrition. In this manner, observes the author, take place inflammation of the eyes from section of the fifth nerve; inflammation of maimed limbs and joints; and the inflammation of typhus. (?)

Inflammation, again, may originate in the blood, when this fluid is so constituted as to give rise to stoppage in the capillary vessels, and thereby to hyperemia and inflammation. This form of the affection may be illustrated by artificial injections into the veins of slimy fluids, as gum-water, potato-starch, or mixtures which contain globules greater than those of human blood, as that of various animals, or quicksilver. The blood may also become more abounding than it ought to be and naturally is with fibrine and albumen; and hence, the circulation in the capillaries may be impeded, and in parts totally obstructed. Globules of pus, mixing with the blood, may produce this untoward effect. And here it may be remarked, that the fact of the globules of pus being greater than those of the blood, and unable consequently to pass the same capillary canals, furnishes an irrefragable objection to the theory of those who suppose that metastatic discharges of a purulent character may take place through the kidney, bowels, &c., without local inflammation of the mucous surfaces of those parts. Further, the blood, from being in particular states of dyscrasia, may contain particles which excite the vascular and nervous tissues, and, acting specifically on the latter, thereby beget inflammation.

All organs and parts are not equally liable to inflammation. The following is the scale of the order of liability of the various tissues to this form of morbid action: cellular tissue, skin, mucous membrane, serous membrane, lungs, liver, and the other parenchymatous glands; then muscle, the great blood-vessels, the nerves, the synovial membranes; lastly, and most rarely, tendons, ligaments, cartilage, bone. Schönlein is of opinion that parts, in which the arterial predominate over the venous vessels, are the more liable.

At page 80, the author treats of what he calls the suppurative plastic stage. Pus, he observes, (p. 87,) is not a mere educt from the blood, nor is it formed from the conversion of any single element of that fluid. It contains, on the contrary, all the chemical constituents of the latter; and the resemblance between the two is so great, that severe suppuration produces precisely the same effects as excessive venesection, while precisely the same means which improve the condition of the blood produce a similar effect on that of the pus.

Pus operates on organic tissues in three ways: 1st. Mechanically, according to the ordinary hydrostatical laws. In this way take place infiltrations into parts and abscesses, and discharges from points far distant from the original seat of disease. 2dly. It operates chemically; macerating simply (if it be healthy pus) the tissues it is in contact with; corroding them if it be ichorous or acrid. Many structural changes are wholly owing to purulent maceration. 3dly. Pus may operate in what Dr. Canstatt calls a vital manner. In this way variolous, syphilitic, vaccinic, scabious varieties of pus produce their respective contagions, and give rise to homologous morbid processes in the parts to which they are applied. This effect cannot be referred to any particular physical or chemical properties of those varieties of pus, but to specific vital action.

The therapeutics of inflammation are discussed at page 91 et seq.; and although the statements and rules are perhaps necessarily and advisedly of a somewhat general and theoretical character, and not much illustrated by particular applications, still various able, acute, and scientific directions and cautions are given, and a variety of important principles established. Our limits do not at all permit us to do more than refer very generally to this part of the work. We shall quote the following observations, partly as a specimen of the author's style of speculative discussion, partly as containing an intelligent, though not entirely novel exposition of the general objects and principles of treatment in inflammation, and in some measure in all diseases:

"In fulfilling this indication, (namely, the general therapeutic one in reference to inflammatory reaction,) what is it we propose? We seek to place the diseased organ and the organism on such a footing as shall enable their vegetative and conservative properties to operate as easily and efficiently as possible, to develope their powers, and compensate, by an act of self-reparation, for any disturbing or alterative influence of the morbid agent. It is not we or our measures that remove anomalous action. The art and the physician who accomplish this is the organism itself. All that we effect is to liberate the normal action from any oppressing or obstructing cause, and afford it freedom of exertion.

"Disease, an abnormal stimulus, itself calls forth this reaction of the organism. The reflexes of the nervous and vascular systems, which are roused by normal stimuli, giving rise to normal reaction, the same reflexes or reactions are opposed by the organism to the alterative influence of the morbid agent. These reflexes are now more and now less diffused; sometimes, in a certain sense, local, sometimes general; affect, now the nervous and vascular systems, now one of these in particular; are, accordingly, sometimes a fever, sometimes a spasm, sometimes complications of both, (as, if you will, in nervous fever.) These reactions may be continued, may be typical. They arise from excitement of the vital power, the tumultuous action produced in which varies according to the nature of the morbid excitant, according to individual constitution, according to the character of external influences, &c. This excitement of the vital power, if it reacts more directly on the affected organ, is of itself often able to recall, and that promptly, the organ to its normal action, and to annihilate the disease. On this ground it is that fever has, by some, been regarded as a salutary process instituted by nature. But these reactions may on the other hand, by their excess, by operating on enfeebled organs, cause the most disastrous consequences, as cerebral and pulmonary hemorrhage, &c., and may therefore with equal propriety be denominated, occasionally, destructive processes instituted by nature. The very same powers and faculties of the organism, which maintain the body in its healthy action, are those which, on the occurrence of disease, become available in offering resistance to that process.

"These high reactions are what first attract the physician's notice. They are often all that he can detect or study concerning the disease. On the other hand, what calls them forth, or against what they are directed, is often obscure and concealed, the physician but recognizing striking reactions in this or that nerve, in this or that part of the vascular system, reactions which concentrate themselves on some particular organ or organs, or else involve the whole chain of organs in an unusual excitement—he may erroneously confound the reaction with the original morbid excitement. And to be sure, reactions may readily become causes of disease.

"On the judgment which he forms of these reactions, are founded the therapeutical measures of the physician. These reactions or reflexes, even when of an unusual kind, are still manifestations of the vital power. In what cases, then, shall we leave them uninterfered with? In what cases shall we venture to oppose them? In what cases shall we endeavour to subdue them, in order that disease may not be grafted on disease? In what cases shall we promote and cooperate with them? Do we propose to leave them free, we follow an expectative method of treatment. Do we chose to withstand them, we act on a sedative plan. Do we think it proper to promote reaction, we stimulate. According as the reaction is general or limited (local?) must be our measures of stimulation or sedation. According as the reaction extends particularly to the nervous or vascular department, so must sedation and stimulation. Hence, every organ, every system has its appropriate nervous and vascular *stimulantia et sedativa.*

"Can the right management of these various modes of treatment be set forth in rules? We think not. This is, of all others, the field for that dangerous arbitrary mode of treatment, which we must place and be content to place under what is called 'practical tact.' Certainly, experience leads us more and more to put faith in the mysterious language of natural instinct; and happy must he be reckoned whose mind is fitted to catch these direct suggestions at the bedside, the accuracy of the interpretation of which can often be made evident by the success of the result only, not by any appreciable process of rational deduction." (pp. 93 4, note.)

It is now generally admitted that many of the seemingly violent phenomena of inflammation are not, strictly speaking, morbid movements, but consist, at least in part, simply of energetic endeavours (reactions) of nature to oppose or rid itself of an injurious agent or influence. As Dr. Canstatt well observes, death may in this way incidentally occur from salutary efforts or intentions of nature itself, as when hemorrhagic apoplexy of the brain or lungs ensues from the reaction instituted to repel or extrude some morbid agent or influence operating on these organs or elsewhere.

The author is at pains to point out the varieties of inflammation, and to lay down rules to guide us as to when depletion is or is not indicated. Against sthenic reaction he considers, as we might expect, bloodletting the "cardinal means." He prefers, on what we think trivial grounds, venesection to arteriotomy. One of his objections to the latter is, that

Schönlein has observed abscess of the brain after opening of the temporal artery! An untoward accident so extremely rare need not, in our opinion, deter us from opening the temporal artery, when this measure is otherwise indicated, any more than the result of Dupuytren's case, in which air entered the vein should frighten us from undertaking any surgical operation, in which section of principal veins is necessary. The one contingency is not more frequent than the other, and both are to be disregarded. Moreover, were all the cases of fatal phlebitis following venesection to be set off against the accident dreaded by Schönlein, we apprehend that the odds would lie against the former in an almost incalculable proportion. In our own experience we have never met with the least inconvenience from temporal arteriotomy.

As anti-plastics against the adhesive tendencies of inflammation, he recommends mercury, nitre, and the alkalies. Dr. Alison, of Edinburgh, has lately on more than one occasion expressed doubts as to whether mercury, administered so as to affect the gums, possesses any power of controlling inflammation and its consequences. In his article on Inflammation in the Library of Medicine, (vol. i. p. 111,) he observes: "Do the symptoms of inflammatory diseases subside more rapidly and more certainly when the mercury has affected the mouth, than without that occurrence? and on this point the present writer cannot hesitate, after considerable experience, to reiterate an opinion he formerly expressed, that he has more frequently seen them aggravated or transferred to another part, on that event taking place, than relieved; and that the cases in which that combination has always seemed to him most useful have been those in which the symptoms having subsided, it was withdrawn without the mouth being affected." We would ask Dr. Alison whether, in the event of the symptoms *not* having so subsided, an issue exceedingly possible, he would not have judged it proper to continue the mercury even till the mouth was affected, because what other assurance could he have that the mineral had touched the system? In our opinion Dr. Alison's statements on this occasion are inconsiderate and extreme; but without taking time for the present to show this, we content ourselves with recommending him to consult the testimonies of several or most of his coadjutors in the very work from which we have made the above quotation, and he will find their statements in regard to the utility of mercury in inflammation, and the propriety of pushing it until the gums are affected, nearly diametrically opposite to his own. We refer him to a few only of these authorities. (Library of Medicine, vol. i. p. 355; vol. ii. pp. 56, 302; vol. iv. p. 146.)

Anomalies of the Secretions. We turned to this chapter with eager curiosity, and regretted to find it less ample and interesting than we expected. Yet, although it contains nothing positively new, it presents, in a concise view, most of the facts and doctrines connected with the subject. Those lesions of secretion are principally treated of, which manifest themselves by mere excess of *hyperkrinie*, ("a barbarous term," as Krantz designates it, introduced by Andral,) or by qualitative degenerations.

In considering the effects of anomalous secretions on the organism, the author points out that the first result of excited secretive action in any particular organ, is to "concentrate" on that organ the secretive energy and activity of the body in general, thereby enfeebling the eliminatory processes elsewhere. Thus, dryness of the skin and constipation accompany diabetes; the urinary secretion fails when profuse sweatings takes place. But this antagonism characterizes only a particular and that an early stage. Long-continued profluvia of every description lead ultimately, by inducing hydremic, pyemic, or other morbid states of the blood, to profluvia of other kinds, to colliquative sweats, to diarrhœa, to hectic with suppuration, &c. Sometimes, in consequence of excessive discharge of one element of the blood, this fluid is left nearly utterly destitute of that particular constituent, as we see happen in the serous discharges of cholera, by which the blood is rendered almost wholly fribinous. But, on the other hand, a particular element of the blood seems sometimes but to augment in proportion as it is excessively secreted or eliminated. This occurs in some cases of albuminous urine, in which the blood is found surcharged with albumen, notwithstanding the abundant exit of it by the bladder.

HETERO-PLASTIC FORMATIONS. At p. 191 there is a short section on this subject. Matter abnormally deposited in the parenchyma of organs becomes gradually solid by coagulation and "crystallization," its watery constituent being absorbed. It then either remains unaltered in the parenchyma when it has been deposited; or it augments by "apposition" of similar matter; or it is changed by the local reaction of the tissues with which it is in contact; or it becomes enlarged by a process of increase within itself, to which Dr. Canstatt gives the name of "intus-susceptive" growth.

The difference between growth by apposition and intus-susception is nosologically important. The former characterizes inorganic, the latter, organic formations. Tubercles increase by apposition merely, not by cellular development, which mode of enlargement is pathognomonic of the pseudo-plastic formations, as cancer, fungus, &c.

Cellular formation is the type of all organic growth; and a previous condition to this formation is the existence of a formative matter, or a cyto-blastema, susceptible of development. The cyto-blastema, in other words, the cystic germ or germinal cyst, has a character either of differency or indifferency. In the latter case, it preserves unchanged the same nature and qualities with which it was secreted or excreted into the parenchyma, presenting, as inspected by the eye, naked or assisted by the microscope, a homogeneous aspect. The differential germinal cyst or formation is, on the other hand, characterized by attracting from the blood materials homologous to itself, and by increasing by the intus-susceptive process. It is of a benign or *euplastic* (ευ and πλασμα) character, when contiguous parts are able to oppose, by pressure, simply its further extension. It is of a *kakoplastic* nature when, like carcinomatous growths, it betrays a tendency to gain ground on all neighbouring tissues, and convert them into a morbid constitution, malignant like its own.

TUBERCULOSIS AND SCROPHULOSIS. According to the author, these affections are identical. Tuberculous matter is deposited either on free membranes or in parenchyma. When in the former situation, as on the mucous membrane of the lungs or bowels, it may escape observation, being liable to be expectorated or evacuated. Kühn professes to have observed in the sputa of phthisical patients the molecules of tubercle; and Magendie says they have been noticed even in the blood of persons strongly predisposed. This we for the present doubt. Canstatt is of opinion that Carswell is in error in supposing the extremities of the mucous ramifications in the lungs to be the exclusive seat of tuberculous deposit in these organs, since in many cases, the minutest air-cells have been found patent and free. Gendrin denies *in toto* the matter in question to be tuberculous, as Carswell and Alison assert it to be; and affirms it to be nothing else than the usual discharge from an inflamed mucous membrane.

Tubercle, examined microscopically, is an amorphous granular mass, wanting, as we have already remarked, the cellular structure of the cytoblastema, the rudimentary form of all organized tissue, wanting also the faculty of intus-suceptive growth by eccentric development. Our readers are familiar with the appearance and characters of tubercle, and with all the facts connected with its various modes of deposition—now miliary and isolated; now confluent in granular masses, or in berry-like or coral-like clusters, as in the lungs; now infiltrated into parenchyma; now drop-like, as on the surface of serous membrane; now cylindrical or branch-like, as in the vessels of the liver. As regards the chemical constitution of tubercle, the greatest difference of opinion still prevails among the most eminent authorities, such as Berzelius, Thenard, Dulong, Lombard, Lassaigne, Hecht, Preuss, and various others. The author would account for this contrariety on the ground, not merely that organo-chemical analysis has not yet reached an adequate degree of technical precision, but that the chemical constitution of tubercle is absolutely not always the same, but modified according to the seat of the deposit, as whether in the lungs, brain, liver, or renal cavities; according to the cachexy leading to the tuberculous development, whether arthritic, puerperal, exanthemic, (p. 207;) lastly, according to the stage of the tubercles themselves. All, he thinks, that we at present satisfactorily know is that, while crude tubercles contain a great proportion of glairy matter, hardened tubercles abound with salts. Thus Lombard found in crude tubercle, of glairy animal matter 98 parts; of salts 1·85 ditto. In chalky tubercle, of animal matter 3 parts; of salts 96 ditto.

It is worthy of remark that tubercle often gives rise to irritation of the parenchyma in its neighbourhood, in consequence of which exudation takes place; which condensing, the tubercle becomes surrounded with something like a cyst. Some have mistaken this cyst for a component part of tubercle; but its origin and history are as we have now stated.

According to Rokitanski, the condition of the blood which leads to or is produced by certain diseases is incompatible with the tuberculous diathesis. These diseases are cancer, and all maladies which give a cyanotic character to the blood, as hypertrophy of the heart; the continued patency of the foramen ovale; emphysema of the lungs; aneurism of the aorta; affections of the spine; typhus; cholera; and dysentery.

We have already stated that the author regards tuberculosis and scrophulosis as identical. The principal ground on which he does so is that, physically, chemically, microscopically tested, the morbid formations characteristic of these are indistinguishable from each other. He answers the objection that scrophulosis is a disease of infancy, tuberculosis of puberty and manhood, by urging that these different periods of life merely determine the development of the disease in somewhat different forms and in different organs; but that, in fact, the scrofulosis of infancy is but tuberculosis of puberty and the contrary. "Is dropsy," he asks, "aught else than dropsy, although in infancy it occurs in the head, in adult age in the belly?" (p. 220.)

Dr. Canstatt's theory, as to the formation of tubercle, is wisely general, and appears to be quite as plausible as those of Andral, Chomel, Louis, Carswell, Alison, or Williams; from some of which it differs slightly or not at all. After stating (p. 222) that "the most of the symptoms of the disease seem to indicate a morbid condition of the fluids (diathesis tuberculosa) which leads to a faulty state of the solids (habitus tuberculosus)," but that, "when we seek to carry our explanations further and to explain in what that degeneracy consists, we find ourselves at a loss"—he adds, "perhaps to be more minute, we may say that the soft formative element (the plasma) destined to supply the solid nutrient constituents of the tissues is produced from a degenerative formative substance (in the albumen?) and does not suffice to supply a mature and normal plasma (fibrine); that hence, the albuminous proportion of the fluids preponderates so much, that at length, the vehicle (the serum) of that albumen is no longer able to hold it in suspension, and it is, in consequence, deposited in the form of tuberculous matter." This was the theory of the late Dr. Todd of Brighton; and Dr. Williams, in speaking of a "degraded state" of the fluids and of degraded and inorganizable depositions, as resulting from a lowered vigour of the system, and from the exhausting effects of chronic diseases, seems to hold, in part, nearly the same views, (Library of Medicine; Article, Tuberculous Diseases.)

We do not accompany Dr. Canstatt through the therapeutical portion of his chapter on tuberculosis and scrophulosis, ample and interesting though the details be. The treatment recommended is varied and eminently judicious, and always propounded with a strict reference to physiological and pathological principles. We refer the reader to the work itself, which in regard to this in common with the other subjects treated of in it, is as rich in practical as in doctrinal details.

We pass over without remark the sections on Lithiasis, Verminous Generation (ento-zoen-bildung), Malacia, and what we may freely render Ossification (verhärtung), and we come to

FEVER. This, preeminently one of the *quæstiones vexatæ* of pathology, Dr. Canstatt proposes in the present volume to discuss as a general subject, reserving for the genetical or historical division of his work the consideration of it in its special manifestations. Much mutual explanation is, in our opinion, necessary between pathologists, and a clear understanding must be come to as to what is implied in the term *idiopathic*, whether it is to be understood relatively or absolutely, before any satisfactory conclusions can be expected to result from discussions respecting the nature of fever. Dr. Christison's article on fever in the Library of Medicine furnishes a lamentable proof of the justness of this remark, seeing that his formal definition of the nature of fever is contradictory, if not unintelligible. We think it unnecessary to detain our readers for a moment, for the purpose of proving a distinction between fever and inflammation. The simple fact of the intermissions of ague, nothing analogous to which we observe in inflammation, demonstrates the non-identity of the diseases.

The author, near the beginning of the section on fever, cautions us against confounding certain symptoms of excitement with those of true reaction. "When," he observes, (p. 259,) "the optic nerve is pressed upon, there is a momentary perception of light. This is a proof of the effect of the exciting cause, not of reaction." He also regards the pains, spasms, and convulsions, which sometimes accompany fever, as explicable from the laws of irradiation and reflexion, and as not essential constituents of the disease. He illustrates reaction somewhat mechanically, by the property which enables a body originally endowed with a certain degree of elasticity to resume, when elongated by any force, its natural dimensions. "In the organism," he remarks, "there is a vital endeavour resembling the elasticity now described." With deference to Dr. Canstatt, we cannot forbear remarking that the illustration adds not a tittle to our knowledge of the subject in question, to wit, the power of vital reaction. What we name, *in concreto*, disease, he thinks is to be regarded as consisting of a series of morbid influences or movements tending to overcome the vital elasticity on the one hand, and efforts of reaction on nature's part on the other.

The usual divisions of fever are adopted, namely, into the stages of invasion, reaction, and crisis; or the period at which either the disease or the reaction finally predominates. The stage of invasion is, of course, sometimes so violent, as in cases of plague, cholera, death by lightning, that there is no subsequent one. The prognosis in the stage of reaction is bad when there is a striking discrepancy between the objective and subjective symptoms; as, for example, when the patient with a dry tongue feels no thirst, with a cold skin complains of heat or the contrary; and when all looks for the worst, thinks himself well and boasts of his strength.

In discoursing of the crisis, Dr. Canstatt justly, we think, refuses his assent to the notion that the discharges, which occasionally signalize the accession of this stage, constitute or contain the *materia peccans*, the presence of which in the system has caused the morbid commotion. He explains these discharges as the mere consequences of the crisis; or, in other words, as simple incidental proofs that the organs have assumed their suspended or impeded functions and actions. Strictly and correctly speaking, the crisis itself is this recovery, or, as the author calls it, this regeneration of the functions; the discharges being nothing but at once the effects and the proofs of that regeneration.

The author's account of what he calls the "rhythm" of fevers is curious, and we believe original. He attributes the exacerbations in febrile diseases, not as is usually done, to fresh ebullitions as it were of the morbid process, but to bolder and more vigorous manifestations of the reactive energy of the system; and accordingly he accounts for a fever being a continued one, by supposing that the reaction is, in this case, constant and sustained. The theory does not certainly solve all difficulties, but deserves attention, and, at any rate, indicates ingenuity.

The causes of fevers are very briefly discussed. (pp. 271-2.) With regard to the question whether there be or be not such an entity as essential fever, we think Dr. Canstatt, in his very concise remarks, states, and that with singular precision, all that can be satisfactorily, for the present at least, laid down on the subject.

"There is" he observes, "no fever, without previous alteration of the organism, in some one or other of its constituents, fluid or solid. Essential fever which comes not under this definition is a non-entity. It can, however, happen that the organism, thus fevered, may so resist the alteration of the blood (as in exanthemic or miasmatic cases) that no fixed or localized disease of solid parts shall ensue. In this case, the fever (or rather the alteration of the blood connected with it) is primary as regards such local disease, yet not primary in itself, but is always an effect; always secondary to that primary vitiation of the blood," &c.

Want of space prevents us from accompanying the author in his Treatment of Fever; but the reader will find Dr. Canstatt's views on this important matter repay the trouble of examination.

THE NEUROSES. It is not our design to give anything like a thorough digest of this part, nor is it necessary. Much of the information which it contains is to be found elsewhere, namely, in the writings of Romberg, Rey, Müller, Sachs, Henle; and in various English works—the principal contents of which, however, are derived from the above sources, or from other continental productions. The translation of Müller's Elements of Physiology, by Dr. Baly, offers to those not familiar with the language of the author the means of becoming acquainted with the most recent, authentic, and philosophical doctrinal views in neuro-physiology, and, to a considerable extent, in neuro-pathology. Referring the reader, therefore, to these and other sources of information, we shall confine ourselves to merely partial notices of Dr. Canstatt's chapter on the Neuroses.

After an exordium, in which the author refers to the rapid progress which neuro-physiology is making in comparison with its "sister" science, neuro-pathology, he proceeds to lay down, first, the laws of nervous irradiation, or the transmission, by the medium of the cerebro-spinal axis, of any irritation of one nerve to another homologous nerve—as of a motor to a motor nerve: and, then, the laws of nervous reflex, or the transmission by the same circuit, namely, the cerebrospinal axis, of any such irritation of a nerve to other but heterologous nerves—as from a motor to sensitive or trophical nerves. Our readers are familiar with these laws.

In respect of their origin or seat, Dr. Canstatt divides neurotic affections into central, peripheric, and synergic. In the first of these, the morbid cause is seated in the nervous centres; and we are generally able to recognize lesions in this situation by the affection being radiated over a variety of nerves taking their origins contiguously in the brain, but distributed on the opposite side from that on which the pain is felt. Affections of cerebral origin have, moreover, no intermission, but are continued. A neurotic affection, again, of peripheral origin, as from a swelled ganglion, or a wound, is distinguishable by being confined to a particular tract or nerve, or if it does give rise to irradiative or reflex actions, these take place on the same side with the primary affection. Synergic neurotic affections are just those of irradiation or reflex, corresponding almost exactly with lesions involving the excito-motory phenomena of Dr. Hall, and are very various; comprehending blepharospasm, laryngismus stridulus, asthma, hiccup, vomiting, tenesmus, strangury, hydrophobia, chorea, hysteria, tetanus, epilepsy. Several of these are remarkable for making their attack during the first sleep. Some are so gradual that the brain has time to watch their approach from the periphery, before the irradiative or reflex effect takes place. This is seen in the case of the *aura epileptica,* and *globus hystericus.* It is to be remarked, however, that the *aura* is sometimes also perceived when the cause of the epileptic affection is seated in the brain itself, or is " centric," to use the language of Dr. Hall.

The preceding division is founded on the modes of origin of neurotic affection. The classification, according to *kind,* proposed by the author, is as follows:

| | |
|---|---|
| Sensible<br>Motory<br>Trophical<br>Intelligential | Neuroses. |

These he further subdivides:

| | |
|---|---|
| Erethism of Sensible Nerves . . | Algies* (Hyperesthesies.) |
| Paralysis ditto . . | Anesthesies. |
| Erethism of Motor Nerves . . | Spasm. |
| Paralysis ditto . . | Paralysis. |
| Erethism of Trophical Nerves . . | Nervous or Erethismal Congestion, Hemorrhage, Secretion. |
| Erethism of the Psychical Nervous Province . . . . . . | Psychical Anesthesies—Imbecility. |
| Pathological Idiosyncrasies. | |

Our physical acquaintance with the trophical department of the nervous system is extremely limited. As the author justly observes, we are still at a loss to determine whether the sympathetic should be viewed as derived from, or as merely sending branches to the spinal and cerebral nerves, with which it communicates. The complete mixing also of the nervous filaments in the ganglia of the sympathetic will probably baffle, for ever, all efforts to gain a knowledge of the structure and physiology of the nerve. From the fact that parts, the sensible nerves of which pass through the sympathetic, have less sensibility than those parts, the nerves of which communicate directly with the spinal system, he is disposed to look favorably on Reid's theory of the sympathetic ganglia being somewhat of the nature of non-conductors or isolators. But Müller and other physiologists explain this phenomenon differently.

Dr. Canstatt regards a natural " high temperature" or constitutional facility of being excited by stimuli as peculiarly predisposing to neurotic affections, (p. 290;) and from the page now referred to on to page 295, we have an interesting sketch of other predisposing causes, cosmical, telluric, climatic, atmospheric, dietetic. Reference is made (p. 294) to those forms of nervous affection which have a " psychological contagious" origin, as chorea, hysteria, epilepsy, induced in excitable persons by the mere sight of others suffering from these disorders. Neurotic affections, such as the algies and spasms, are said by Dr.

* We use this word for want of a better; and as the corresponding translation in English to the German " Algie," employed by the author.

Canstatt to affect the right side more particularly; lesions of anesthesy and paralysis, the left. But this requires confirmation.

Some interesting observations follow, on the "rhythm" of neurotic affections. The excitability of the nervous system becomes exhausted under long stimulation of any sort, and sinks into a species of sub-paralysis; during which, the same exciting cause does not produce the same reaction, as at first. But by and bye, when the organism has re-accumulated, during this dormancy, excitability and power, a new manifestation of reaction takes place. This is illustrated when any swelling, as a node on a bone, presses on a sensible nerve. The node may remain undiminished in size, but the pain intermits. The sympathetic, it is well known, differs from other nerves, by the circumstance of the rhythm of its sensibility being apt to continue a long time after the exciting cause has been withdrawn. In some respects, all nervous affections are apt, if allowed to continue, to acquire what the author fancifully calls a burgher right, (bürger-recht.) The epilepsy, strabismus, amaurosis, induced by the intestinal irritation of worms, may remain after the expulsion of the original cause.

With regard to the crises of certain neurotic affections, the author cautions us not to consider the vomiting of blood or the melæna with which neuralgia cœliaca sometimes terminates, or the diarrhœa which occasionally marks the disappearance of a psychical affection, and other like coincidences, as essentially connected with the more prominent nervous lesion. These may merely indicate the withdrawal of the morbid cause which gave rise simultaneously to the neurotic and coincident derangement, whether diarrhœa, hemorrhage, vomiting; and the resumption, on the part of the organism, of its wonted vigour and regularity. No more is the sleep, in the course of which many neuroses take their leave, to be viewed as the crisis. It, *in common* with the cessation of the nervous disorder, is the effect of the crisis, not the crisis itself; two things very distinct.

An affection of one part of the nervous system is apt to extend to other parts. Thus peripheral neuroses tend to become central also: a long-continued peripheral neuralgy may and often does affect the psychical department of the brain, giving rise to delirium or mania. So also hallucinations, in consequence of hyperesthesies of the nerves of sense, have become at length profoundly psychological. Hysteria, occasionally, merges into nymphomania; epilepsy, into idiotcy; all which successions of secondary on their respective primary affections illustrate the law above laid down. So also amaurosis of one eye is frequently followed by that of its fellow. This propagation of lesion takes place more readily from the centre to the circumference than the reverse.

From the reflex property of the cerebrospinal nerves, we receive an explanation of (to adopt the language of the old physicians) the progression of a neurotic affection, *sine materia*, into one, *cum materia.* This results from reflex actions of the cerebrospinal on the organic, vaso-motory, or trophical nerves. Every paroxysm of an erethismal neurosis is accompanied by vascular symptoms: the part which is the seat of the neuralgy becomes injected; its arteries pulsate more strongly; it swells; its secretion is increased, (if it be a secreting organ or surface:)

these changes and the consequent derangement of the capillaries of the part lead to plastic alteration, to hypertrophy, to softening or to hardening.

The prognosis in nervous diseases is concisely but acutely stated. Neurotic affections are so apt to become rhythmical, that the longer they continue the more gloomy is the prospect of their entire and final removal. Those, of course, which depend on structural changes, are the most hopeless. Peripheral are more curable than central neuroses; those of which the causes are apparent and easily removable than those of an opposite sort. Thus convulsions from worms present a fair prospect of cure. Erethismal neuroses are more tractable than those of an anæsthetical character. Spasmodic affections are more serious than neuralgic. Certain neurotic maladies, as tetanus, hydrophobia, &c. are, from the circumstances which attend them, essentially dangerous; but hysteria, hypochondria, and even epilepsy, are not incompatible with reasonably long life. The tendency of the neuroses to pass into inflammation, or into structural change, as pointed out in the preceding paragraph, is always to be kept in mind.

We do not accompany Dr. Canstatt in his therapeutic observations. He seems to place great faith in the metallic sedatives, the salts of zinc, bismuth, copper, silver, iron, arsenic: and states that, of these, zinc, copper, silver, as well as the alkalies, are more adapted for lesions of the motory nerves; bismuth, iron, and arsenic, for lesions of the sensible nerves. As "notoriously" sedative, he recommends Artemisia root. He is very sceptical as to the advantages to be expected from revulsives, employed with the view of diverting the nervous excitement from one locality to another, since he thinks that the facility of intercommunion between all parts of the nervous system makes it as likely that the revulsive shall augment the morbid excitement as drive it to another organ or part.

But our limits forbid us to prosecute further an examination of this volume; and we deem this the less necessary, as in his handling of the special neuroses, to wit, the neuralgies, spasmodic affections, anesthesies, akinesies, and the psychoses, the author follows, in a great measure, the principles laid down in regard to the neuroses considered as a class, and of which we have endeavoured to give the reader a partial idea.

We have thus gone through, in the cursory manner we proposed, Dr. Canstatt's able production. Our opinion of its merits is so fully implied in the course and manner of our analysis, that it is not needful to enlarge on this subject. We regard it as a work considerably above mediocrity; as very careful in its statements of facts; as scientific in its deductions from these, as well as in its general views and reasonings. As Falstaff was, on his own report, not only witty in himself, but, moreover, the cause of wit in others; so Dr. Canstatt is not only an intelligent and thoughtful writer, but has the art and merit of giving rise to trains of useful reflection in his reader. His speculation rarely exceeds the legitimate bounds of probability, and is in general based on authentic and substantial physiological and pathological data. The author evinces a sufficient degree of professional reading and erudition, and appears familiar with British medical literature, both of an earlier and a more recent date.

The chief defect of the work is an occasional vagueness both of expression and idea, which, it cannot be denied, is a common fault of German authors, even when handling scientific and practical subjects. We look with interest for the second part of this work; since favorable as is our opinion of the author as a merely practical writer, we think it likely that he will show himself to be still better qualified for the more abstruse and theoretical discussions, to which that branch of his subject will necessarily lead.

---

